# Recurrent *BRCA1* Mutation, but no *BRCA2* Mutation, in Vietnamese Patients with Ovarian Carcinoma Detected with Next Generation Sequencing

**DOI:** 10.31557/APJCP.2020.21.8.2331

**Published:** 2020-08

**Authors:** Hoang Anh Vu, Ngo Dai Phu, Le Thai Khuong, Pham Huy Hoa, Bui Thi Hong Nhu, Vo Thanh Nhan, Le Quang Thanh, Nguyen Duy Sinh, Hoang Thanh Chi, Nguyen Dang Quan, Nguyen Trong Binh

**Affiliations:** 1 *Center for Molecular Biomedicine, University of Medicine and Pharmacy at Ho Chi Minh City, Ho Chi Minh City, Vietnam. *; 2 *University of Science - Vietnam National University Ho Chi Minh City, Ho Chi Minh City, Vietnam. *; 3 *Tu Du Hospital, Ho Chi Minh City, Vietnam. *; 4 *Vinmec Central Park International Hospital, Ho Chi Minh City, Vietnam. *; 5 *Mekophar Chemical Pharmaceutical Joint Stock Company, Ho Chi Minh City, Vietnam. *; 6 *Biotechnology Center of Ho Chi Minh City, Ho Chi Minh City, Vietnam. *

**Keywords:** Ovarian carcinoma, BRCA1, BRCA2, Vietnamese

## Abstract

**Background:**

Identification of germline and somatic *BRCA1/2* mutations in ovarian cancer is important for genetic counseling and treatment decision making with poly ADP ribose polymerase inhibitors. Unfortunately, data on the frequency of *BRCA1/2* mutations in Vietnamese patients are scare.

**Methods:**

We aim to explore the occurrence of *BRCA1/2 *mutations in 101 Vietnamese patients with ovarian cancer including serous (n = 58), endometrioid (n = 14), mucinous (n = 24), and clear cell (n = 5) carcinomas. *BRCA1/2* mutations were detected from formalin-fixed parafin-embedded tumor samples using the Oncomine^TM^ BRCA Research Assay on Personal Genome Machine Platform with Ion Reporter Software for sequencing data analysis. The presence of pathogenic mutations was confirmed by Sanger sequencing.

**Results:**

We found no *BRCA2* mutation in the entire cohort. Four types of pathogenic mutations in *BRCA1 *(Ser454Ter, Gln541Ter, Arg1751Ter, and Gln1779AsnfsTer14) were detected in 8 unrelated patients (7.9%) belonging to serous and endometrioid carcinoma groups. Except for the c.1360_1361delAG (Ser454Ter) mutation in *BRCA1 *exon 11 that was somatic, the other mutations in exons 11, 20, and 22 were germline. Interestingly, the recurrent Arg1751Ter mutation in *BRCA1* exon 20 appeared in 4 patients, suggesting that this is a founder mutation in Vietnamese patients.

**Conclusion:**

Mutational analysis of tumor tissue using next generation sequencing allowed the detection of both germline and somatic *BRCA1/2* mutations.

## Introduction

Ovarian cancer, which encompasses a heterogeneous group of malignancies, is a relatively rare disease with a high case-fatality rate. Globally, there are 295,414 new diagnoses and 184,799deaths from the disease each year (Bray et al., 2018). More than 90% of ovarian cancers are epithelial, with the most common being serous carcinoma. Even though the general population lifetime risk of ovarian cancer is only approximately 1.4%, individuals at high-risk of developing the disease due to harboring a germline *BRCA1* and *BRCA2* mutation have an average cumulative risk of between 40% to 75% and 11% to 34%, respectively (Mavaddat et al., 2013).

Surgery, followed by chemotherapy regimens based on platinum salts, is still the standard of care in ovarian cancer (Narod, 2016). Since 2014, indications of poly ADP ribose polymerase (PARP) inhibitors became available for ovarian cancer patients with germline or somatic mutations in *BRCA1*and *BRCA2* genes (Ledermann et al., 2014). Therefore, tumor *BRCA1/2* testing is a powerful tool to identify mutations in ovarian cancer patients which have been shown to benefit from treatment with PARP inhibitors (Lheureux et al., 2017; Vergote et al., 2016).

Because the *BRCA1/2* genes lack hot spot mutations, it is essential to sequence the complete coding regions and intron/exon junctions to determine the mutation status of *BRCA1*or *BRCA2*. Although Sanger sequencing is traditionally used for detection of *BRCA1/2* mutations in clinical samples, next generation sequencing (NGS) has recently allowed obtaining a complete coverage of all exonic regions. This is very crucial because *BRCA *mutations differ among patients of different ethnicity (Kim et al., 2016). NGS-based tumor testing has the advantage of identification of both germline and somatic mutations (Weren et al., 2017).

About 1,500 new patients with ovarian cancer and 856 deaths was reported in Vietnam during 2018 (Bray et al., 2018). In this study, through mutational analysis of both genes from 101tumor tissues, we established the frequency and type of *BRCA1/2* mutations in Vietnamese patients with ovarian cancer.

## Materials and Methods

*Patients and samples*

Samples for the study were collected from 101 unrelated patients with ovarian carcinoma, diagnosed by using standard histological criteria, at the Tu Du Hospital, Ho Chi Minh City, Vietnam. Samples included 46 FFPE and blood-matched samples and 55 FFPE-only samples (corresponding normal FFPE samples were used as paired controls for specimens that carried pathogenic mutations). Of the 101 samples, 58 were serous carcinoma, 24 were mucinous carcinoma, 14 were endometrioid carcinoma, and 5 were clear cell carcinoma. The study was approved by the Ethics Committee of University of Medicine and Pharmacy at Ho Chi Minh City. All subjects were counseled and provided written informed consent for the study.

*DNA extraction and UDG treatment*

Tumor-rich areas, which contained at least 50% of the tumor cells on a hematoxylin and eosin slide, were marked by the pathologist (PHH), micro-dissected using a 21G needle. For enzymatic removal of cytosine deamination artifacts with uracil-DNA-glycosylate (UDG), genomic DNA was extracted from three 10 µm-thick FFPE sections using the GeneRead DNA FFPE kit (Qiagen, Hilden, Germany) according to the manufacturer’s protocol. In selected samples, genomic DNA was extracted fromperipheral blood leukocytes using the QIAamp DNA Mini Kit (Qiagen, Hilden, Germany). Quantity and quality of isolated DNA was determined with a NanoDrop 2000 (Thermo Scientific, MA, USA) and a Qubit 4.0 fluorometer (Thermo Scientific, Waltham, MA, USA).

*BRCA1/2 sequencing and data processing with NGS*

Two positive controls containing somatic and germline variants with known allele frequencies were used in parallel with patients’ samples. The somatic positive control was BRCA Somatic Multiplex I gDNA and the germline positive control was BRCA Germline I gDNA (Horizon, Cambridge, UK). Genomic DNA (10 ng) was amplified using 265 primerpairs in two pools (OncomineTMBRCA Research Assay, Thermo Fisher Scientific) according to the manufacturer’s protocol on Personal Genome Machine (PGM) (Thermo Fisher Scientific, Carlsbad, CA, USA). Amplicons were ligated to a barcode adaptor usingIon XpressBarcode Adapters 1- 16Kit (Life Technologies, Carlsbad, CA, USA). We then enriched the barcoded library by emulsion PCR using Ion PGM™ Hi-Q™ View OT2 Kit (Thermo Fisher Scientific) on OneTouch2 and OneTouch ES instruments (Life Technologies). We sequenced the enriched library using the Ion Torrent PGM platform with an Ion 318TM chip (Life Technologies). The mean sequencing depth for FFPE tumor samples was 2,625x, with a mean uniformity of 94.4%. Sequencing data analysis was performed using Torrent Suite version 5.0.5 and Ion Reporter version 5.6 (Thermo Fisher Scientific). Variants with a read count < 50 and a variant frequency < 10% were not analyzed further (Ivanov et at., 2017). The annotation is based on the *BRCA1* transcript ENSG00000012048 (NM_007294.3) and the *BRCA2 *transcript ENSG00000139618 (NM_000059.3). Variants were classified as pathogenic if they were well-known, previously reported (based on CLINVAR and COSMIC database) or they were frameshift or stop mutations.

Pathogenic mutations have been confirmed by Sanger sequencing, using the BigDye Terminator v3.1 sequencing kit and the ABI PRISM 3500 Genetic Analyzer (Life Technologies). Finally, for determination of the somatic or germline nature of detected mutations in 8 *BRCA1* positive tumor samples, DNA isolated from non-neoplastic cells was amplified by PCR followed by Sanger sequencing.

*Statistical analysis*

The relationship between *BRCA* mutations and individual variables were analyzed with the use of *χ*^2^/Fisher’s exact test. Statistical analyses were performed using SPSS software. Statistical significance was defined as P-value less than 0.05

## Results

*Analytic workflow*

**Figure 1:**
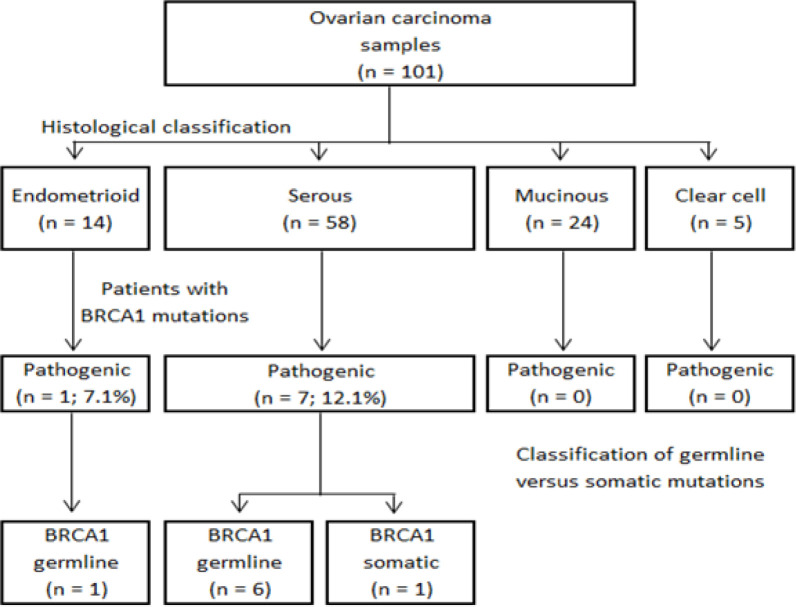
*BRCA1/2* Genetic Mutations Identified in the Study. Distribution of pathogenic *BRCA1/2* mutations according to different histological subtypes of ovarian carcinoma

A total of 101 cases of ovarian carcinoma samples were analyzed. The histological subtypes included serous (n = 58), endometrioid (n = 14), mucinous (n = 24), and clear cell (n = 5) carcinomas. Figure 1 shows the workflow for the identification of *BRCA1/2* pathogenic mutations in the current study. The Figure 1 illustrates that *BRCA* mutations appeared in serous and endometrioid carcinomas but not in mucinous and clear cell carcinomas.

*Detection of BRCA1 and BRCA2 mutations by NGS and Sanger sequencing*

We was able to confirm all the *BRCA* variants from both positive controls in the current study with NGS technique. Then, data analysis from patients’ NGS has documented 7 pathogenic variants, with allele frequency > 10%, in 11 patients. These variants included 5 in the *BRCA1* gene (c.1360_1361delAG, c.1621C>T, c.3352C>T, c.5251C>T, and c.5335delC) and 2 in *BRCA2* gene (c.9117G>A and c.4366G>T). Sanger sequencing from the corresponding patients’ tumor DNA failed to confirm any of the 2 variants in *BRCA2* as well as the c.3352C>T variant in *BRCA1*. Therefore, we concluded that NGS gave rise to 3 false-positive mutations in this cohort. Of the Sanger-confirmed pathogenic *BRCA1* mutations in 8 patients, c.5251C>T appeared in 4 unrelated patients, c.1621C>T in 2 unrelated patients; while each of the c.1360_1361delAG and c.5335delC appeared in a single patient (Table 1).

**Figure 2:**
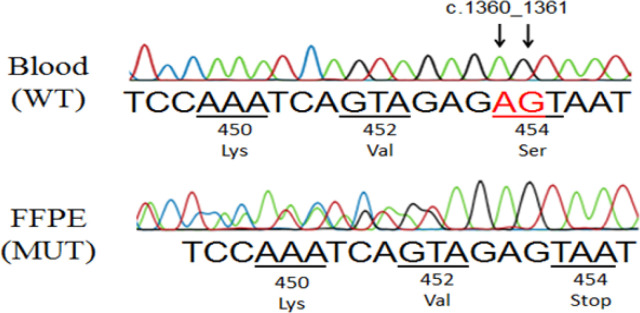
Confirmation of the *BRCA1* Somatic Mutation in OCY16T Sample. Sanger sequencing analysis in reverse direction shown that the c.1360_1361delAG on *BRCA1* exon 11 was present in FFPE sample (below), but not in the blood sample (above).

In order to clarify whether each of the *BRCA1* mutations was germline or somatic, Sanger sequencing of normal samples (blood samples or normal FFPE samples) from corresponding patients was performed. In the Figure 2, Sanger analysis showed that the c.1360_1361delAG mutation in patient OCY16T was not present in normal cells, thus confirmed somatic nature of this mutant. In the remaining 7 patients, *BRCA1* mutations were germline (figure not shown).

Demographic and clinical characteristics of the patients are shown in Table 2. All mutations occurred in serous or endometrioid carcinomas, but not in mucinous and clear cell carcinomas. 

**Table 1:** Pathogenic Mutations in the *BRCA1* Gene Detected in 8 Unrelated Patients

Sample ID	Exon	Nucleotide change	Amino acid change	Read depth (X)	Variant frequency (%)	Germline (G)/ Somatic (S)	Histology	Age (years)
HGSOC12	22	c.5335delC	p.Gln1779AsnfsTer14	438	38	G	Serous carcinoma, grade 3	48
HGSOC26	20	c.5251C>T	p.Arg1751Ter	1773	63	G	Serous carcinoma, grade 2	48
HGSOC37	20	c.5251C>T	p.Arg1751Ter	1881	90	G	Serous carcinoma, grade 2	51
HGSOC45	11	c.1621C>T	p.Gln541Ter	1997	88	G	Serous carcinoma, grade 2	51
OCY16T	11	c.1360_1361delAG	p.Ser454Ter	1990	60	S	Serous carcinoma, grade 3	54
OCY23T	20	c.5251C>T	p.Arg1751Ter	1525	70	G	Endometrioid carcinoma, grade 3	50
OCY33T	11	c.1621C>T	p.Gln541Ter	1365	93	G	Serous carcinoma, grade 2	53
OCY41T	20	c.5251C>T	p.Arg1751Ter	993	51	G	Serous carcinoma, grade 1	49

**Table 2:** Comparison of Demographic and Clinical Characteristics of 101 Patients According to *BRCA *Mutation Status

Characteristic		No. of patients (N = 101)	BRCA1 mutation		*P*-value of univariate analysis
			
			Positive	Negative	
			(n = 8)	(n = 93)	
Age (year)	Median	49.7	50.5	49.6	0.786
	Range	18 – 75	48 – 54	18 – 75	
Age group, No. (%)	≤ 50 years	53	4 (7.5%)	49 (92.5%)	0.858
	> 50 years	48	4 (8.3%)	44 (91.7%)	
Histological subtype	Serous	58	7 (12.1%)	51 (87.9%)	0.004
of ovarian carcinoma, No. (%)	Endometrioid	14	1 (7.1%)	13 (92.9%)	
	Mucinous	24	0 (0 %)	24 (100.0%)	
	Clear cell	5	0 (0 %)	05 (100.0%)	

## Discussion

Detection of mutations in the *BRCA1* and *BRCA2 *genes from ovarian cancer is meaningful for both genetic counseling and treatment decision making with PARP inhibitors. Mutational analysis of tumor DNA can determine germline and somatic mutations, which is suitable for both purposes. However, it is nearly impossible to use traditional Sanger sequencing for investigation of the entire coding region of the two genes using FFPE tissue samples, due to the large size of these genes and the fragmentation of DNA from FFPE material. Here, we demonstrated that NGS was able to survey the entire coding region of *BRCA1/2*. Besides the 3 false-positive cases, which we could not explain thoroughly, NGS correctly detected *BRCA1* mutations in 8 out of 101 patients (7.9%). The frequency of *BRCA1* and *BRCA 2* mutations in ovarian cancer varied greatly in previous reports, ranging from 1.1 to 39.7% and 0 to 13.9%, respectively (Shanmughapriya et al., 2013). We did not find any *BRCA2* mutation, quite similar to reports from Hungary (Van der Looij et al., 2000) and Sweden (Einbeigi et al., 2007). *BRCA2* mutations in ovarian cancer are indeed so rare in several countries (0.9 - 2.5%) like Finland (Sarantaus et al., 2001), Pakistan (Liede et al., 2002), India (Vaidyanathan et al., 2009), and Denmark (Soegaard et al., 2008). Our result suggested that the *BRCA2* gene might not play a considerable role in pathogenesis of ovarian cancer in Vietnamese patients.

Previous studies have shown that mucinous ovarian carcinomas did not carry *BRCA1/2* mutations (Capoluongo et al., 2018). In agreement with these, we did not detect any mutations of *BRCA1* or *BRCA2* from 24 cases of this histological entity. If we omit the mucinous ovarian carcinoma group from entire cohort, the rate of *BRCA1* mutation in Vietnamese patients with ovarian carcinoma should be 10.4% (8 out of 77 patients). Also, in agreement with previous reports (Mavaddat et al., 2012), we detected most *BRCA1* mutations from serous carcinomas (7 out of 58 patients; 12.1%) and with lower frequency from endometrioid carcinomas (1 out of 14 patients; 7.1%).

The most interesting point in our study was that up to 4 patients (50% in the mutant group) carried the same mutation in *BRCA1* exon 20. This recurrent mutation (c.5251C> T, p.Arg1751Ter) has been reported in 3 Vietnamese patients in a study of 200 cases of Asian-origin breast cancer patients living in the USA (Kurian et al., 2008). Whether this is a founder mutation predisposing ovarian cancer in Vietnamese needs to be studied with larger numbers of subject. Some founder mutations of *BRCA1/2* have been recorded in many different races (Janavičius et al., 2010). Particularly, the p.Arg1751Ter mutation has been recognized as founder mutation of Greek, Hungarian, Middle Eastern, Italian and Polish patients (Bu et al., 2016; Kowalik et al., 2018).

In conclusion, this is the first study in Vietnam to use NGS to investigate mutations of *BRCA1* and *BRCA2 *genes in ovarian cancer patients. Although the *BRCA2* gene mutation was not documented, we found that 10.4% of patients with ovarian carcinoma other than mucinous carcinoma carry the *BRCA1* gene mutation. Finally, the *BRCA1 p.Arg1751Ter* mutation may be a founder mutation in Vietnamese patients.

## References

[B1] Bray F, Ferlay J, Soerjomataram I (2018). Global cancer statistics 2018: GLOBOCAN estimates of incidence and mortality worldwide for 36 cancers in 185 countries. CA Cancer J Clin.

[B2] Bu R, Siraj AK, Al-Obaisi KA (2016). Identification of novel BRCA founder mutations in Middle Eastern breast cancer patients using capture and Sanger sequencing analysis. Int J Cancer.

[B3] Capoluongo E, Scambia G, Nabholtz JM (2018). Main implications related to the switch to BRCA1/2 tumor testing in ovarian cancer patients: A proposal of a consensus. Oncotarget.

[B4] Einbeigi Z, Bergman A, Meis-Kindblom JM (2007). Occurrence of both breast and ovarian cancer in a woman is a marker for the BRCA gene mutations: a population-based study from western Sweden. Fam Cancer.

[B5] Ivanov M, Laktionov K, Breder V (2017). Towards standardization of next-generation sequencing of FFPE samples for clinical oncology: intrinsic obstacles and possible solutions. J Transl Med.

[B6] Janavičius R (2010). Founder BRCA1/2 mutations in the Europe: implications for hereditary breast-ovarian cancer prevention and control. EPMA J.

[B7] Kim YC, Zhao L, Zhang H (2016). Prevalence and spectrum of BRCA germline variants in mainland Chinese familial breast and ovarian cancer patients. Oncotarget.

[B8] Kowalik A, Siołek M, Kopczyński J (2018). BRCA1 founder mutations and beyond in the Polish population: A single-institution BRCA1/2 next-generation sequencing study. PLoS One.

[B9] Kurian AW, Gong GD, Chun NM (2008). Performance of BRCA1/2 mutation prediction models in Asian Americans. J Clin Oncol.

[B10] Ledermann J, Harter P, Gourley C (2014). Olaparib maintenance therapy in patients with platinum-sensitive relapsed serous ovarian cancer: a preplanned retrospective analysis of outcomes by BRCA status in a randomised phase 2 trial. Lancet Oncol.

[B11] Lheureux S, Lai Z, Dougherty BA (2017). Long-term responders on olaparib maintenance in high-grade serous ovarian cancer: clinical and molecular characterization. Clin Cancer Res.

[B12] Liede A, Malik IA, Aziz Z (2002). Contribution of BRCA1 and BRCA2 mutations to breast and ovarian cancer in Pakistan. Am J Hum Genet.

[B13] Mavaddat N, Barrowdale D, Andrulis I (2012). Consortium of investigators of modifiers of BRCA1/2 Pathology of breast and ovarian cancers among BRCA1 and BRCA2 mutation carriers: results from the Consortium of Investigators of Modifiers of BRCA1/2 (CIMBA). Cancer Epidemiol Biomarkers Prev.

[B14] Mavaddat N, Peock S, Frost D (2013). Cancer risks for BRCA1 and BRCA2 mutation carriers: results from prospective analysis of EMBRACE. J Nat Cancer Institut.

[B15] Narod S (2016). Can advanced-stage ovarian cancer be cured?. Nat Rev Clin Oncol.

[B16] Sarantaus L, Vahteristo P, Bloom E (2001). BRCA1 and BRCA2 mutations among 233 unselected Finnish ovarian carcinoma patients. Eur J Hum Genet.

[B17] Shanmughapriya S, Nachiappan V, Natarajaseenivasan K (2013). BRCA1 and BRCA2 mutations in the ovarian cancer population across race and ethnicity: special reference to Asia. Oncology.

[B18] Soegaard M, Kjaer SK, Cox M (2008). BRCA1 and BRCA2 mutation prevalence and clinical characteristics of a population-based series of ovarian cancer cases from Denmark. Clin Cancer Res.

[B19] Vaidyanathan K, Lakhotia S, Ravishankar HM (2009). BRCA1 and BRCA2 germline mutation analysis among Indian women from south India: identification of four novel mutations and high-frequency occurrence of 185delAG mutation. J Biosci.

[B20] Van der Looij M, Szabo C, Besznyak I (2000). Prevalence of founder BRCA1 and BRCA2 mutations among breast and ovarian cancer patients in Hungary. Int J Cancer.

[B21] Vergote I, Banerjee S, Gerdes AM (2016). Current perspectives on recommendations for BRCA genetic testing in ovarian cancer patients. Eur J Cancer.

[B22] Weren RD, Mensenkamp AR, Simons M (2017). Novel BRCA1 and BRCA2 tumor test as basis for treatment decisions and referral for genetic counselling of patients with ovarian carcinomas. Hum Mutat.

